# Low failure rate by means of DLBP fixation of undisplaced femoral neck fractures

**DOI:** 10.1007/s00068-016-0659-4

**Published:** 2016-03-19

**Authors:** A. D. P. van Walsum, J. Vroemen, H. M. J. Janzing, T. Winkelhorst, J. Kalsbeek, W. H. Roerdink

**Affiliations:** 1 Department of Trauma Surgery, Medisch Spectrum Twente, Koningsplein 1, 7512 KZ Enschede, The Netherlands; 2 Department of Trauma Surgery, Amphia Ziekenhuis, Molengracht 21, 4818 CK Breda, The Netherlands; 3 Department of Trauma Surgery, VieCuri Medical Centre, Tegelseweg 210, 5912 BL Venlo, The Netherlands; 4 Department of Trauma Surgery, Canisius Wilhelmina, Weg door Jonkerbos 100, 6532 SZ Nijmegen, The Netherlands; 5 Department of Trauma Surgery, Deventer Ziekenhuis, Nico Bolkesteinlaan 75, 7416 SE Deventer, The Netherlands

**Keywords:** Femoral neck fracture, Intracapsular hip fracture, Hip fracture, Undisplaced, Non displaced, Internal fixation, Osteosynthesis, Avascular necrosis, Rotational stability

## Abstract

**Background:**

This study evaluated the clinical results of a new implant in the internal fixation of undisplaced femoral neck fractures.

**Method:**

Irrespective of their age, 149 patients with undisplaced (Garden I and II) femoral neck fractures were included in a prospective multicentre clinical cohort study and were treated by internal fixation by means of the Dynamic Locking Blade Plate (DLBP). The mean age was 69 years and the follow-up at least one year.

**Results:**

The DLBP fixation resulted in 6 out of 149 failures caused by AVN (2x), non-union (2x), loss of fixation (3x) or combination of these.

**Conclusion:**

The fixation of undisplaced femoral neck fractures by the DLBP resulted in a low failure rate of 4 %.

## Introduction

The biology plays a leading role in the survival of the femoral head and the bone healing of this intracapsular fracture. Critical biological factors are the (re)-vascularisation of the femoral head and the type of bone healing of the femoral neck fractures. The viability of the femoral head after a femoral neck fracture is dependent on preservation of the remaining vascularity and on revascularisation and repair of the necrotic areas before collapse of the necrotic bone segment can occur. Although the vascularisation of the femoral head in the undisplaced fracture is less damaged than in the displaced fractures, the incidence of avascular necrosis for undisplaced femoral neck fractures is 4.0 versus 9.5 % for the displaced fractures [1]. To preserve the remaining vascularisation of the femoral head we must do no further vascular harm during insertion of implants in the head of femur. Therefore, any iatrogenic fracture displacement should be avoided, especially rotation of the femoral head on insertion of our implants. One of the sources of revascularisation of the femoral head is the vascular ingrowth across the uniting fracture line. It is of clinical importance that these ingrowing tender vascular buds can be torn repeatedly if there is persistent motion at the fracture site as a result of inadequate fracture stabilisation [2]. Enlarging the volume of metal in the femoral head may further compromise the revascularisation of the femoral head and this may increase the incidence of avascular necrosis [3, 4]. Unlike diaphyseal fractures, the femoral neck fracture cannot heal by periosteal (external) callus formation. Consequently, the bone healing is by primary osteonal reconstruction that requires an anatomical reduction and absolute stability [5–7]. Only when the undisplaced femoral neck fracture is secured by stable fixation, revascularisation of the femoral head can take place and the fracture can heal by primary osteonal reconstruction. The term “stable”, in the context of fixation of femoral neck fractures, means that transverse shear- and the rotational inter fragmentary movements (IFM) are minimalized while allowing the controlled axial compression IFM.

Approximately 20 % of the intracapsular hip fractures are undisplaced [12]. Common treatment is internal fixation of the fracture, but alternative treatments are conservative treatment or replacement arthroplasty. The conventional implants used for the fixation of femoral neck fractures are the sliding hip screw devices and multiple parallel screws or pins. The failure rate after internal fixation of undisplaced femoral neck fractures is 8–14 % [8–13]. The potential disadvantages of the conventional implants are rotational and/or angular instability combined with a relative high implant volume in the femoral head [14]. The aim of this study was to register the results in the internal fixation of undisplaced femoral neck fractures by means of the DLBP. This device is characterised by angular and rotational stability, dynamic compression and a low implant volume in the head of femur.

## Patients and methods

### Classification

According the conventional Garden classification an undisplaced intracapsular fracture is defined as Garden grade I or II fracture. This classification is based only on the AP radiograph and includes all fractures impacted into any degree of valgus (Garden I) and the undisplaced fractures (Garden II). Consequently, also the fractures that show angulation on the lateral radiograph are included and classified as undisplaced.

### Patients

Included were undisplaced femoral neck fractures in adult patients irrespective the age of the patient. Excluded were pathological fractures, concomitant fractures of the lower extremity, symptomatic arthritis, local infection or inflammation, inadequate local tissue coverage, morbid obesity and any mental or neuromuscular disorder, which would create an unacceptable risk of fixation failure or complications in postoperative care.

### Implant

**Fig. 1:**
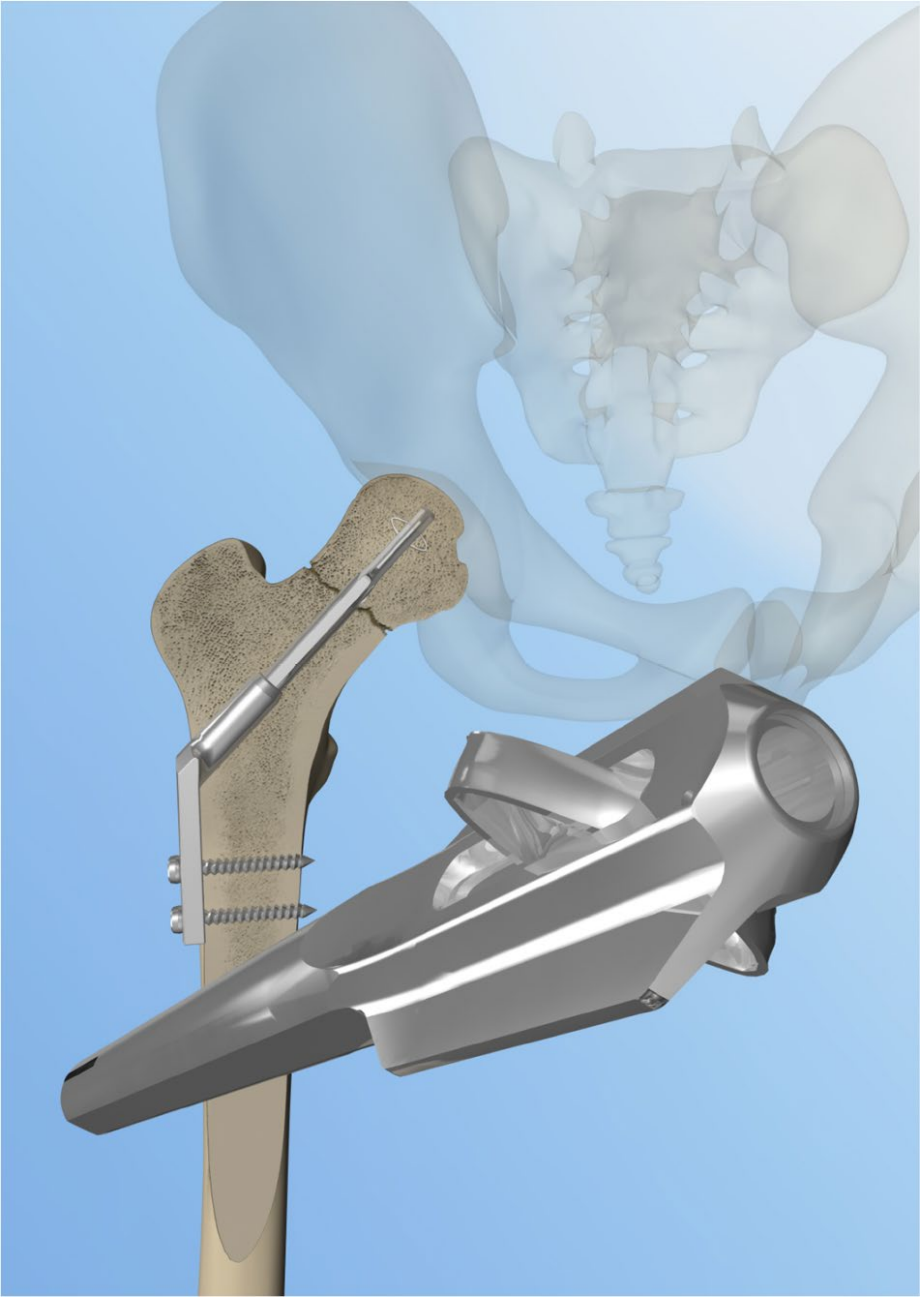
Design of the dynamic locking blade plate

The DLBP consists of a 2-hole standard 135° side-plate combined with a low-volume cannulated dynamic locking blade. The side plate provides angular stability combined with dynamic axial compression of the fracture. Two side wings at the tip of the blade provide rotational stable fixation of the locking blade in the femoral head combined with a high weight-bearing surface. The expandable impaction anchors lock the blade in the femoral head and prevent perforation and backing out of the implant and further augment the rotational stability. The DLBP is now marketed as the Gannet (Fig. 1).

### Technique

**Fig. 2:**
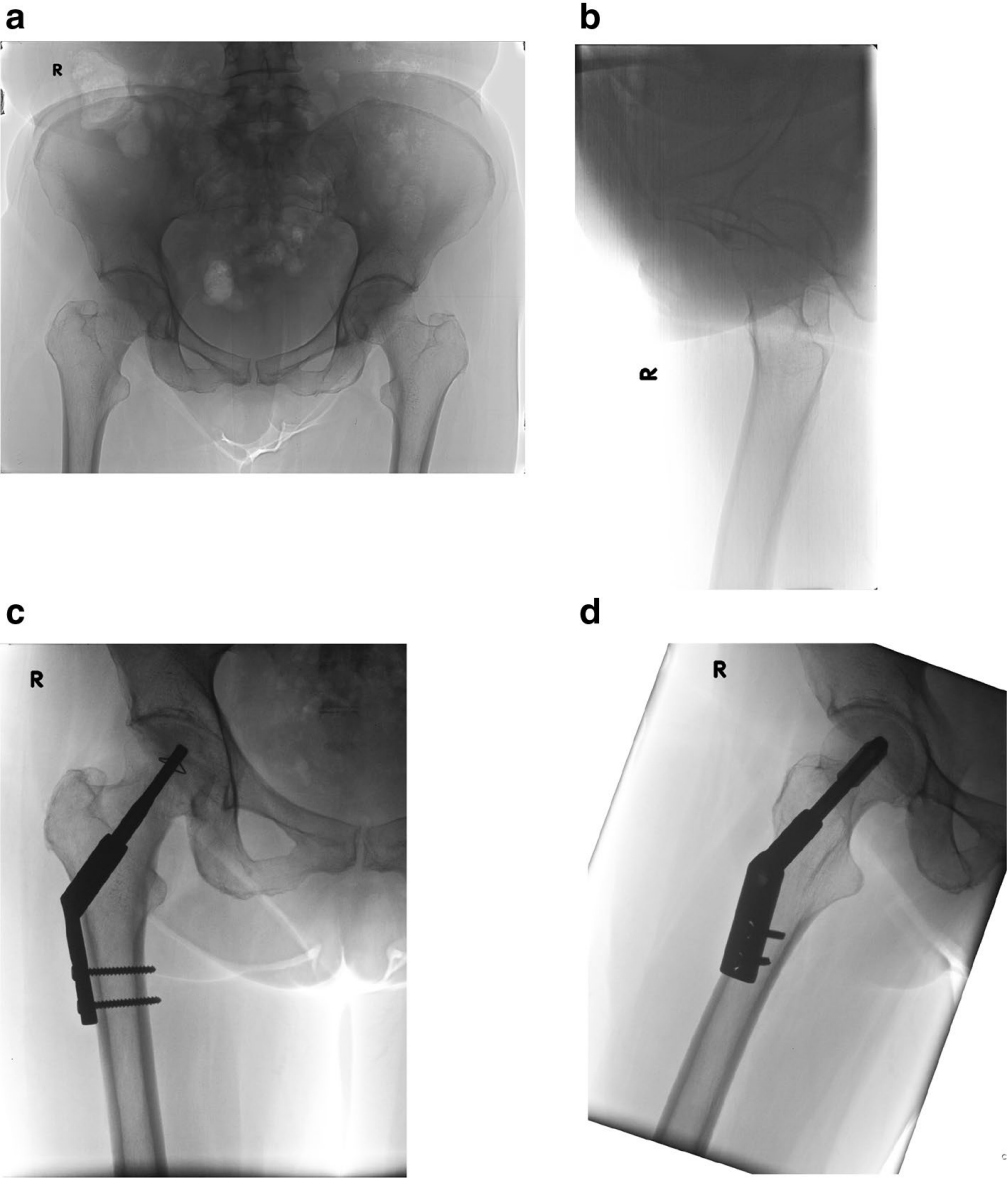
**a**, **b** AP en lateral X-ray of undisplaced femoral neck fracture of the right hip, **c**, **d** AP and lateral X-ray of undisplaced femoral neck fracture of the right hip after DLBP fixation

If there is any (anterior) angulation of the femoral neck with dorsal displacement of the femoral head, anatomical reduction is performed by internal rotation and anterior manual compression. To do no further vascular harm, the reduction should be performed gently and accurately as excessive longitudinal traction and rotation may result in additional vascular damage by tearing the still surviving retinacular vessels. By a ±7 cm lateral approach a 3.0-mm 135° guide wire is placed in the centre/centre position in femoral head. After length measuring cannulated reaming is performed up to 5 mm subchondrally in the femoral head. Next the locking blade together with a two-hole side plate is mounted on the introducer. The complete implant is introduced over the guide wire and gently tapped in while the mounted side plate functions as a rotational guide. After the side plate is seated along the lateral cortex, the introducer is released and the locking blade further tapped in the femoral head up to 5 mm subchondrally. Next, the side plate is fixed to the proximal femur by two self-tapping cortical screws. By turning the setscrew, in the shaft of the locking blade in clockwise direction, the impaction anchors are expanded by which the blade is locked within the femoral head. On removal, turning the setscrew anti clockwise retracts the impaction anchors. After removal of the cortical screws, the locking blade together with the side plate is tapped out by means of an extractor mounted on the locking blade. The patients were mobilised postoperatively by permissive weight bearing as tolerated by the patient. The implant characteristics and operative technique are further illustrated in YouTube video (gannet implant) (Fig. 2).

## Methods

The Garden classification is based on the pre-operative AP radiograph of the hip. The (anterior) angulation of the fracture is assessed on the lateral pre-operative radiograph of the hip. Postoperative AP and lateral radiographs were used to assess fracture healing. Union was defined by an absence of visible margins of the fracture. Angular instability was assessed radiologically by secondary interfragmentary angulation and/or transverse shear. Interfragmentary rotation was radiologically assessed by the observation of a cortical step and diameter mismatch at the fracture site. Non-union was identified by either displacement of the fracture or clearly visible margins of the fracture 1 year postoperatively. Avascular necrosis was defined according to the Steinberg classification from stage 2 and upward [15]. Failure of fixation is defined as the need for revision surgery because of non-union, avascular necrosis or cut out of the implant. The corrected Tip Apex Distance (TAD) on the first postoperative X-rays assessed the position of the locking blade in the femoral head [16]. A TAD greater then 25 mm is predictive of a higher extrusion rate. The impaction at the fracture site was assessed by measuring the degree of telescoping of the dynamic blade with correction for magnification. Mobility was assessed by the need of walking aids: no walking aids, one crutch, two crutches or a walker.

## Results

One Level-1 Community Trauma center (Medische Spectrum Twente, Enschede) and four Level-2 Community teaching hospitals (Deventer Ziekenhuis, Deventer; Amphia Ziekenhuis, Breda; Canisius Wilhelmina Ziekenhuis, Nijmegen and VieCuri Medisch Centrum, Venlo) participated. Between 01-08-2010 and 19-12-2013, and 384 consecutive patients with femoral neck fractures were treated by means of the DLBP. Of these 384 patients, 172 a suffered undisplaced femoral neck fracture and 212 a displaced femoral neck fracture. This manuscript addresses the results of the patients who are treated for undisplaced fractures. 172 patients with undisplaced (Garden I and II) femoral neck fractures were included irrespective of the age of the patient. Seven patients were lost for follow-up and 16 patients died during the follow-up period. This resulted in 149 patients with a mean age of 69 years (35–101) with a follow-up of at least 1 year from injury. Surgery was undertaken by (orthopaedic) trauma surgeons (85 %), and trainee surgeons (15 %). 79 % of operations took place within 24 h. The average operating time was 39 min. There were six general medical complications: one deep infection (healed without intervention surgery, two postoperative bleedings and three pneumonia). Implant-related complications consisted of suboptimal expansion of the impaction anchors in four cases. In two younger patients this was caused by the high bone density, and in two elderly patients by technical implant problems with the expansion mechanism. Neither perforation, nor backing-out of the dynamic blade was observed. No secondary rotational or angular instability was observed. No breakage of the blade, plate or screws occurred. The internal fixation of undisplaced (Garden I and II) femoral neck fractures resulted in 6 out of 149 failures (4.0 %) caused by AVN (2×), non-union (2×), loss of fixation (3×) or combination of these. All of the six failed fixations were revised by arthroplasty. Five of the failures were classified as a Garden I fracture and one Garden II. AVN was observed in two out of six failures (one Garden I, one Garden II). In two out of the six failures anterior angulation (posterior displacement) was more than 20°. The mean impaction of the healed fractures was 5 mm with a mean age of 69 years. The mean age in the failure group was 71 years. The average TAD in the healed fracture group was 22 mm and 24 mm in the failure group.

Elective implant removal was performed in 9 % due to suspected local complaints caused by the side plate or the (dynamized) blade. In all patients the implant removal, including the retraction of the anchors, went straightforward. Four per cent of the patients with healed femoral neck fractures needed more walking aids than before fracture.

## Discussion

The most common treatment of undisplaced femoral neck fractures is internal fixation by sliding hip screw devices or multiple parallel screws or pins. However, alternative treatments are conservative treatment or replacement arthroplasty. A review study by Conn and Parker confirmed a non-union rate of 30–45 % for conservative treatment [11]. The recent review study concluded that the non-union rate with secondary displacement was at least 30 % for the conservative treatment of undisplaced femoral neck fractures [13]. To avoid the complications of avascular necrosis and non-union, arthroplasty was advocated in the treatment of undisplaced femoral neck fractures [17]. However, hemi-arthroplasty is complicated by deep infection (3 %), superficial infection (15 %), periprosthetic fracture (3 %), dislocation (5 %), loosening (10 %), acetabular wear (20 %) and a potentially higher mortality compared to internal fixation [11].

The failure rate after internal fixation of undisplaced femoral neck fractures remains relatively low [8]. Nevertheless internal fixation is not without complications. Parker described 6.4 % non-union, 4.0 % avascular necrosis and revision surgery in 7.7 % [11]. The review study by Van Embden demonstrated a non-union rate of 4–8.5 %, avascular necrosis in 2–4 % and revision surgery of 8–15 % after osteosynthesis of undisplaced femoral neck fractures [13]. The still considerable failure rate after internal fixation of undisplaced femoral neck fractures cannot be solely attributed to the implants. Other factors such as a-traumatic surgical technique and the positioning of the implant are as important as the choice of implant.

In this study the DLBP fixation proved to provide stable fixation of the undisplaced femoral neck fractures with a failure rate of 6 out of 149 (4 %). However, in two of these failed fractures the lateral radiograph showed more than 20° of anterior angulation (32° and 40°). Therefore, it seems controversial if these so-called stable Garden 1 and 2 fractures, with significant anterior angulation (posterior displacement), really behave as stable fractures or should be classified as unstable. If only the Garden I and II fractures with an angulation on lateral imaging of less than 20° were classified as stable, the failure rate in this study would drop from 4.0 to 2.9 %. The stability of the DLBP fixation is further demonstrated by the fact that neither secondary, rotational or angular instability nor perforation or backing-out of the dynamic blade was observed. Furthermore, a TAD greater then 25 mm did not prove to be predictive of a higher extrusion rate as is the case with the standard implants. The vascularity of the head of femur after DLBP fixation is as such that AVN led to failure only in 1.2 % of the included patients. The viability and stability are also apparent from the low degree of fracture impaction with a mean of 5 mm.

The DLBP was designed to follow the biology of the femoral neck fracture. Therefore, the DLBP is a low-volume, dynamic implant, providing angular and rotational stability. The volume (of the proximal 25 mm of the implant in the femoral head) of the DLPB is 1500 mm^3^ compared to 2600 mm^3^ for the DHS and 2800 mm^3^ for DHS spiral blade. The volume of three Asnis screws is 2700 mm^3^. The square diameter of the DLBP is 31 mm^2^ compared to 133 mm^2^ for the DHS/DHS Spiral Blade and 99 mm^2^ for three Asnis screws. The weight-bearing surface of the DLBP is 338 mm^2^ compared with 221 mm^2^ for the DHS. Torsion test showed that the rotational stability of the DLBP triples that of the DHS [14]. The resulting failure rate of the DLBP fixation of the undisplaced femoral neck fractures is low (4 %) and compares favourably with the results of the common implants (8–14 %). The most commonly used implants are the multiple parallel screws or pins and the sliding hip screw devices (SHS) with both comparable results. The potential implant-related factors in the failure rate for the screw fixation are the intrinsic lack of angular and rotational stability. The stability reached is dependent of the exact positioning of the screws and is, therefore, surgeon dependent. The SHS also lacks rotational stability with the added risk of iatrogenic rotation of the head during insertion of the implant [14, 18–20]. Another potential risk factor is the relative high implant volume in the femoral head for the screw fixation and the SHS devices [21]. In the operative treatment of femoral neck fractures minimal invasiveness seems to be more than the length of the skin incision. Probably more important is the minimal invasiveness to the femoral head characterised by a low volume and a low cross section of he implant in the femoral head and neck. The hypothesised advantageous characteristics of the DLBP are the combination of angular and rotational stability and low implant volume. Although the results of the DLBP in this study are promising, we recognise that this observational cohort study is not the strongest study design to prove this. Also it is recognised that functional evaluation was limited.

## Conclusion

Based on the good clinical results, internal fixation seems to be the optimum treatment for the undisplaced femoral neck fracture. However, the failure rate of 8–14 % is still disturbing. Although not all failures are implant-related, the choice of implant plays a role in the final outcome. The possible implant-related factors are the lack of angle and/or rotational stability in combination of a high implant volume in the head of femur. The DLBP (Gannet) was designed to improve the stability of the femoral neck fracture paired to minimal invasiveness to the femoral head. The low failure rate of the DLBP fixation of undisplaced femoral neck fractures of 4.0 % seems to be promising and further supports the treatment algorithm that no effort should be spared to preserve the femoral head after an undisplaced femoral neck fracture by internal fixation irrespective of the age of the patient.
